# Chopsticks-Induced Traumatic Choroidal Rupture With Submacular Haemorrhage in a Child

**DOI:** 10.7759/cureus.112108

**Published:** 2026-07-05

**Authors:** Atiqah Wan Khairuzzaman, Mazaya Mahmud, Jemaima Che Hamzah, Rona Asnida Nasaruddin, Wan Haslina Wan Abdul Halim

**Affiliations:** 1 Department of Ophthalmology, Hospital Universiti Kebangsaan Malaysia, Kuala Lumpur, MYS; 2 Department of Ophthalmology, Universiti Putra Malaysia, Serdang, MYS

**Keywords:** choroidal rupture, ocular trauma, paediatric trauma, submacular haemorrhage, traumatic cataract

## Abstract

We report a case of traumatic choroidal rupture in a child caused by accidental injury with chopsticks at school. A healthy 12-year-old boy presented with severe right eye pain, tearing, and significant blurring of vision (6/60), right eye subconjunctival haemorrhage, corneal abrasion, traumatic mydriasis with uveitis, and posterior subcapsular rosette cataract as well as a massive submacular haemorrhage with 360 degrees of peripheral commotio retinae. Optical coherence tomography of the macula revealed areas of choroidal rupture near the optic disc and at the fovea. Conservative treatment was pursued due to the complexity of paediatric ocular surgery and the risks of intervention. Over time, visual acuity showed limited improvement to 6/36. This case highlights the diagnostic and therapeutic challenges of choroidal rupture in children, the importance of trauma prevention in children, and raises awareness on school-related injuries.

## Introduction

Eye injuries caused by blunt force are prevalent among paediatric cases treated in the emergency department and remain a significant cause of preventable ocular morbidity [1]. Children are particularly vulnerable to these injuries because of their participation in play activities and exposure to household objects and toys. Blunt ocular trauma can result in a spectrum of anterior and posterior segment injuries, including corneal epithelial defects, hyphema, lens-related injuries, commotio retinae, choroidal rupture, retinal tears or detachment, and traumatic optic neuropathy. Although many cases appear to be benign on initial external examination, blunt ocular trauma resulting in severe consequences has been documented in the literature, even from seemingly harmless household items and toys, such as in our case involving chopsticks, resulting in choroidal rupture [2]. This highlights the unpredictable nature of blunt trauma and the potential for significant intraocular damage in the absence of external ocular signs. Furthermore, the diagnosis may be particularly challenging, as children may have difficulty in accurately describing their symptoms or the mechanism of injury, especially when the incident occurs without adult supervision.

This case underscores the critical importance of maintaining a high index of suspicion and performing a comprehensive ocular assessment, including a dilated fundus examination and appropriate imaging when indicated, in all instances of blunt eye injury, regardless of the absence of visible external trauma.

This article was previously presented as a poster at the 13th Conjoint Ophthalmology Scientific Conference in conjunction with the 8th Universiti Sains Malaysia (USM) Ophthalmology Symposium, Kota Bharu, Kelantan, Malaysia, on September 13, 2024.

## Case presentation

A 12-year-old previously healthy boy presented to the emergency department following an accidental blunt injury to his right eye while playing with chopsticks at school. He reported immediate onset of severe ocular pain, excessive tearing, and marked visual blurring in the affected eye.

**Figure 1:**
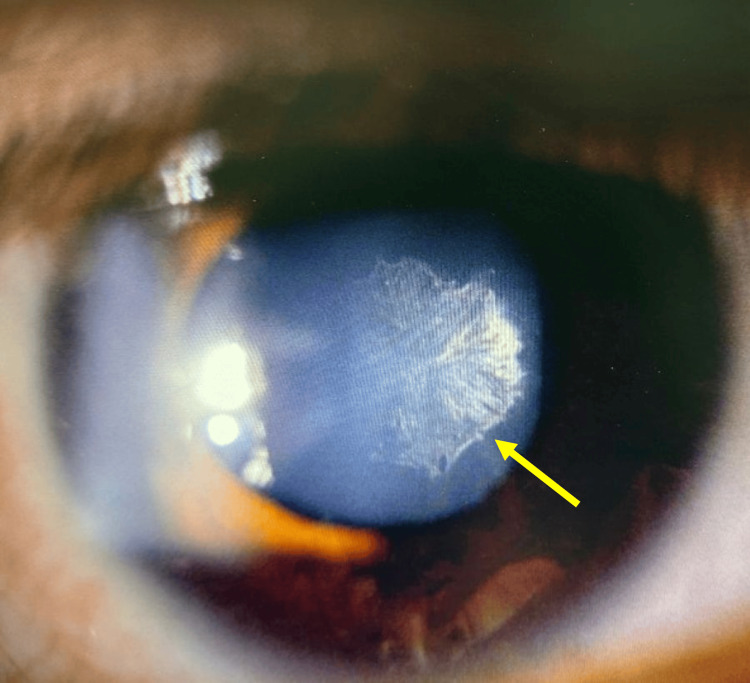
Slit-lamp photograph showing a posterior subcapsular rosette-shaped cataract in the right eye following blunt ocular injury (yellow arrow).

On examination, right eye visual acuity was 6/60, with no relative afferent pupillary defect. External inspection revealed mild subconjunctival haemorrhage and a superficial corneal epithelial abrasion. Anterior segment evaluation showed traumatic mydriasis, anterior uveitis, and a posterior subcapsular rosette-shaped cataract (Figure 1). Intraocular pressure was within normal limits.

**Figure 2:**
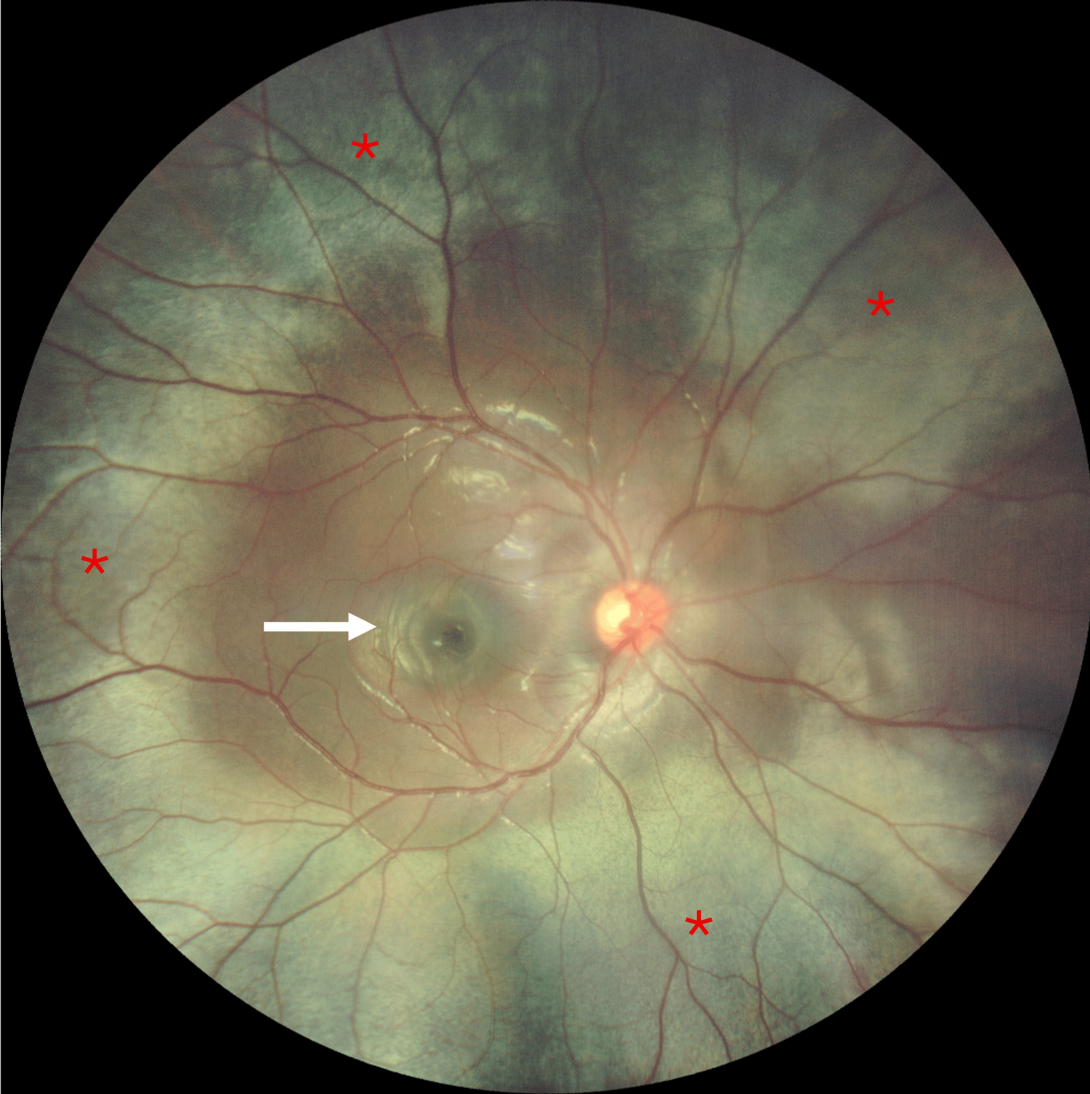
Color fundus image demonstrating extensive 360 degrees peripheral commotio retinae (red asterisks) and a submacular haemorrhage involving the foveal center in the right eye (white arrow).

**Figure 3:**
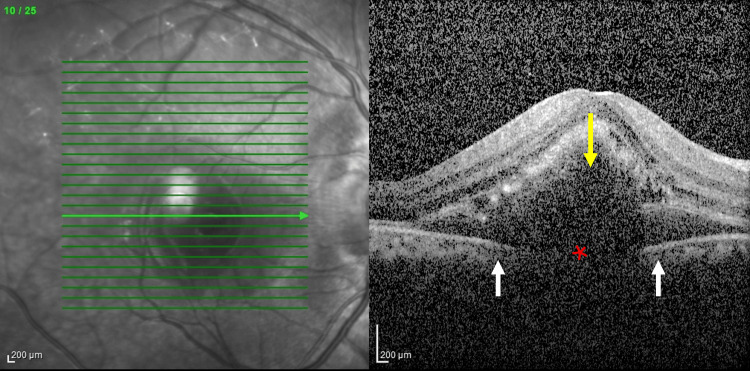
OCT macula of the right eye on presentation revealing a massive submacular haemorrhage (yellow arrow) with choroidal rupture as evidenced by focal discontinuity of the retinal pigment epithelium -- Bruch’s membrane complex (asterisk) and outward deformation of the choroidal contour (white arrows). OCT, optical coherence tomography.

**Figure 4:**
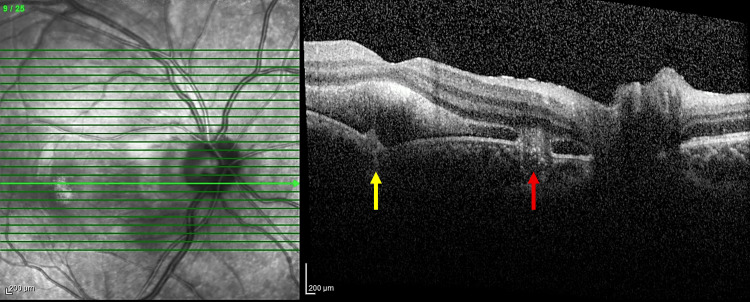
Serial OCT imaging of the right eye at two-week follow-up showing partial resolution of the submacular haemorrhage with more evident choroidal ruptures at the fovea (yellow arrow) and peripapillary region (red arrow). OCT, optical coherence tomography.

Fundus examination revealed a large subretinal haemorrhage involving the macula, along with 360° of peripheral commotio retinae (Figure 2). No retinal breaks or detachments were observed. The external periorbital and ocular adnexal structures were intact, and gonioscopy demonstrated no evidence of angle recession. Optical coherence tomography (OCT) of the macula revealed a large hyperreflective subfoveal lesion corresponding to a massive submacular haemorrhage, with focal discontinuity of the retinal pigment epithelium (RPE) -- Bruch’s membrane complex and outward deformation of the choroidal contour, suggestive of choroidal rupture (Figure 3). Another area of choroidal rupture was also noted near the optic disc. These ruptures became more evident in the OCT macula with gradual resolution of the submacular haemorrhage (Figure 4).

**Figure 5:**
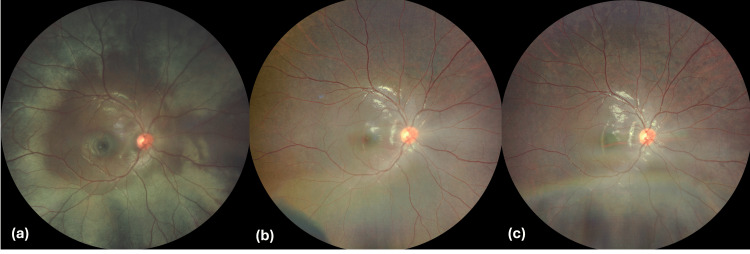
Fundus photographs of the right eye showing submacular haemorrhage and commotio retinae at presentation, (a) with gradual resolution at one-month follow-up (b) and six-month follow-up, (c) demonstrating residual macular changes.

The left eye examination was unremarkable. The patient was managed conservatively with topical corticosteroids and antibiotic eye drops and was followed up weekly. The child experienced a fast recovery of pain symptoms within one week, and gradual resolution of the submacular haemorrhage was observed over time (Figure 5).

**Figure 6:**
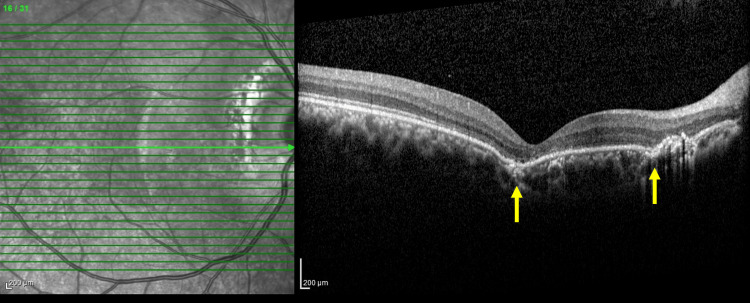
Right eye OCT at six months after trauma demonstrating persistent foveal thinning and outer retinal atrophy associated with a residual concave contour depression at the site of prior ruptures (yellow arrows), correlating with limited visual recovery. OCT, optical coherence tomography.

At six-month follow-up, best corrected visual acuity in the right eye had improved to 6/36, with no evidence of choroidal neovascularisation (CNV) on consecutive visits. However, visual recovery remained limited due to persistent foveal atrophy (Figure 6) and a visually significant traumatic cataract, for which cataract extraction is planned as the definitive treatment, which may offer further improvement in visual acuity.

## Discussion

Choroidal rupture refers to a tear in the choroid, Bruch's membrane, and the RPE, typically resulting from traumatic injury to the eye [3]. While choroidal ruptures are more commonly caused by blunt trauma, they can also occur due to penetrating injuries [4]. In our present case, the trauma was most likely blunt, resulting from the child’s right eye being accidentally struck by the blunt end of a chopstick.

Choroidal rupture may occur more readily in the presence of disorders associated with weakened or brittle Bruch's membrane, whereby even minor trauma can precipitate injury. Such conditions include high myopia, pseudoxanthoma elasticum, Ehlers-Danlos syndrome, and angioid streaks [4]. However, in this case, the child has no identifiable predisposing factors, indicating that the choroidal ruptures were caused solely by the chopsticks injury. These ruptures are associated with several complications that can significantly impair vision, including submacular haemorrhage, and, notably, they carry a risk of late-onset issues such as CNV, which may lead to further vision loss if not properly managed.

In our case, the chopsticks trauma has led to one of the most common complications of choroidal rupture, which is submacular haemorrhage, involving the fovea, which has resolved gradually over time with conservative management, including topical corticosteroids and antibiotic therapy. While visual improvement was modest, which was also attributable to the presence of traumatic posterior subcapsular cataract, the resolution of haemorrhage without the need for surgical intervention was encouraging.

In published studies, rupture location and initial poor visual acuity are independently associated with final visual outcomes. Ruptures occurring closer to the fovea have a greater risk of developing CNV than those situated more than 1500 µm from the foveal avascular zone. Moreover, ruptures measuring around 4000 µm in length are susceptible to neovascularisation regardless of their proximity to the fovea [5]. Although in our case one of the ruptures occurred directly on the fovea, fortunately, the child did not develop this complication, and visual acuity remained unchanged throughout consecutive visits. However, he remains under close surveillance with initial weekly visits and subsequently monthly follow-up visits, including regular dilated fundus examinations and OCT monitoring, to allow early detection and prompt management if CNV develops.

 A recent study by Kim et al. reported visual recovery from 20/50 to 20/20 with resolved subretinal haemorrhage in a 14-year-old boy with choroidal rupture following intravitreal bevacizumab injection [6]. Other non-invasive treatments reported in the literature include intravitreal or subretinal injection of recombinant tissue plasminogen activator alone or in combination with pneumatic displacement, with variable visual prognosis [7]. However, there are multiple case reports in the literature, such as those documented by Adhi and colleagues, that have described instances of spontaneous resolution of traumatic submacular haemorrhage without the need for any medical or surgical intervention [8]. These reports highlight that, in select cases, the haemorrhage may gradually clear on its own over time, leading to partial or even significant visual recovery, although the overall prognosis can vary depending on factors such as the size and location of the haemorrhage, the patient’s age, and the presence of any underlying ocular conditions.

Another key issue to be addressed in this case is the management of traumatic cataracts in the paediatric population, which pose significant challenges in diagnosis, surgical timing, and visual rehabilitation, not only due to the immediate visual impairment they cause but also because of ongoing growth of the eye, long life expectancy, and their potential to lead to amblyopia if not adequately managed [9]. Amblyopia, which may result from deprivation due to cataract-induced visual loss, is a leading cause of preventable monocular visual impairment in children [10]. Although the critical period for amblyopia treatment spans from birth to approximately 8-10 years of age, during which the visual system is highly responsive to corrective interventions, amblyopia can still occur in older children. If left untreated, amblyopia may potentially result in permanent visual deficits, adversely affecting quality of life later in adulthood; thus, definitive treatment would be cataract extraction.

## Conclusions

Blunt ocular trauma is a common presentation, particularly among the paediatric population, and remains an important cause of preventable visual morbidity worldwide. Despite prompt ophthalmic assessment and timely intervention, the visual prognosis may still be guarded, depending on the severity and extent of intraocular damage and on the development of secondary complications such as retinal injury, choroidal rupture, or traumatic cataract. In paediatric patients, the impact may be further compounded by challenges in examination, delayed symptom reporting, and the potential long-term effects on visual development and amblyopia risk.

This case highlights that even seemingly innocuous objects can result in significant ocular injury, underscoring the unpredictable nature of blunt trauma. A high index of suspicion and a comprehensive ophthalmic evaluation, including a dilated fundus examination and appropriate imaging, are essential in all cases, regardless of the absence of external signs. Awareness and education regarding the risks of eye injuries, alongside the implementation of safety measures and supervision protocols in school environments, are crucial in reducing preventable ocular trauma. Strengthening preventive strategies at both community and institutional levels is key to ensuring a safer environment for children and minimising long-term visual disability.
